# Multimodal interaction in the insect brain

**DOI:** 10.1186/s12868-016-0258-7

**Published:** 2016-06-01

**Authors:** Anna Balkenius, Christian Balkenius

**Affiliations:** Department of Plant Protection Biology, Swedish University of Agricultural Sciences, Box 102, 230 53 Alnarp, Sweden; Lund University Cognitive Science, Box 192, 221 00 Lund, Sweden

**Keywords:** Moth, Multmodal interaction, Inverse effeciveness, Superadditivity, Temporal window

## Abstract

****Background**:**

The magnitude of multimodal enhancement in the brain is believed to depend on the stimulus intensity and timing. Such an effect has been found in many species, but has not been previously investigated in insects.

****Results**:**

We investigated the responses to multimodal stimuli consisting of an odour and a colour in the antennal lobe and mushroom body of the moth *Manduca sexta*. The mushroom body shows enhanced responses for multimodal stimuli consisting of a general flower odour and a blue colour. No such effect was seen for a bergamot odour. The enhancement shows an *inverse effectiveness* where the responses to weaker multimodal stimuli are amplified more than those to stronger stimuli. Furthermore, the enhancement depends on the precise timing of the two stimulus components.

****Conclusions**:**

Insect multimodal processing show both the principle of inverse effectiveness and the existence of an optimal temporal window.

**Electronic supplementary material:**

The online version of this article (doi:10.1186/s12868-016-0258-7) contains supplementary material, which is available to authorized users.

## Background

In nature, nearly all stimuli engage multiple senses. They can be seen, heard, smelled and tasted. Consequently, the brain must deal with the complexity of a rich flow of multimodal information. It is well known that information from different sensory channels is combined and integrated in the nervous system. This results in a robust and unified perception of the external world, and provides animals with considerable response flexibility [1, 2].

In humans, behavioural results have showed that tri-modal cues are detected faster and more accurately than bi-modal cues, which, likewise, show advantages over unimodal responses [3]. Moreover, different modalities are processed at different speeds in different parts of the brain [4]. The activity of neural populations that respond to a particular sensory modality can be modulated by a another modality either by being enhanced or depressed [1, 5]. This interaction can radically transform the experience of the stimulus [6] and consequently also influence behaviour.

Until recently, most brain research has focused on one modality at a time, but sensory inputs of different modalities are not processed independently. Cross-modal interactions are probably the rule and not the exception in perception, and the cortical pathways previously thought to be sensory-specific have been found to be modulated by signals from other modalities [7].

In vertebrates, it has been established that multimodal enhancement is stronger with weaker stimulus intensities [1, 8, 9]. This can be seen in both brain recordings and behaviour and is called The principle of inverse effectiveness. Furthermore, multimodal enhancement depends on the timing of the stimuli resulting in a *temporal window of multimodal integration* [1, 9]. The ideal timing that will give the optimal enhancement depends on the stimuli used and their natural timing differences in nature.

To a flower-foraging insect, there are obvious advantages to using both visual and odour cues while searching for nectar. Odours can be detected from a long range, long before a small flower can be seen [10]. On the other hand, visual cues are very useful for the detailed approach of the flower [10, 11]. It would thus be of interest to investigate if the principles above also hold for insects.

In recent years, the knowledge of the insect brain has greatly increased but we still know very little about the interaction between different sensory inputs to the brain. An important area for multimodal interaction appears to be the mushroom bodies (MB). The mushroom bodies are paired, high-order neuropils involved in complex functions such as learning and memory, sensory integration, context recognition and olfactory processing [5, 12–18].

The mushroom body consists of a calyx, pedunculus and two lobes, one medial and one vertical. The calyx houses dendritic branches of Kenyon cells [19] and the pedunculus and lobes contain the axons and terminals of these neurons respectively. The calyx is doubled and concentrically divided into a broad peripheral zone, which receives input from the olfactory system through antennal lobe (AL) projection neurons, and a narrow inner zone, which most likely receives visual input from the optic lobe (consisting of the lamina, medulla and lobula). The MB is thought to encode odours in sparse patterns of activity, and has mostly been studied with odour responses [12]. The size of the MB varies between species, but its organization is similar in the large *Manduca sexta* and the tiny fruit fly [20, 21].

However, recordings of mushroom body efferent neurons have shown that Kenyon cells carry multimodal sensory information [13–17]. In previous studies with multimodal stimuli in the hawkmoth *M. sexta* we have measured interactions between colours and odours [5, 18].

Although multimodal interaction has been known to occur in insects for some time, the principles for such interaction have not been thoroughly studied. Here, we investigate the principles of inverse effectiveness and the existence of a temporal window for multimodal interaction in the brain of *M. sexta*. We use optical imaging techniques to record responses from the antennal lobe and the mushroom body of *M. sexta* during multimodal stimulus presentation using colour and odour.

## Results

Five experiment were performed. The first four investigated the dose-response curves with odour or multimodal stimuli and the third experiment investigated the temporal response window.

### Bergamot processing is not influenced by a visual cue

The first two experiment used bergamot (BM) in different concentrations with or without visual stimulation. The two modalities were presented together for 1 s while the responses of the AL (Experiment 1) or MB (Experiment 2) were recorded from 18 and 21 animals respectively.

**Fig. 1:**
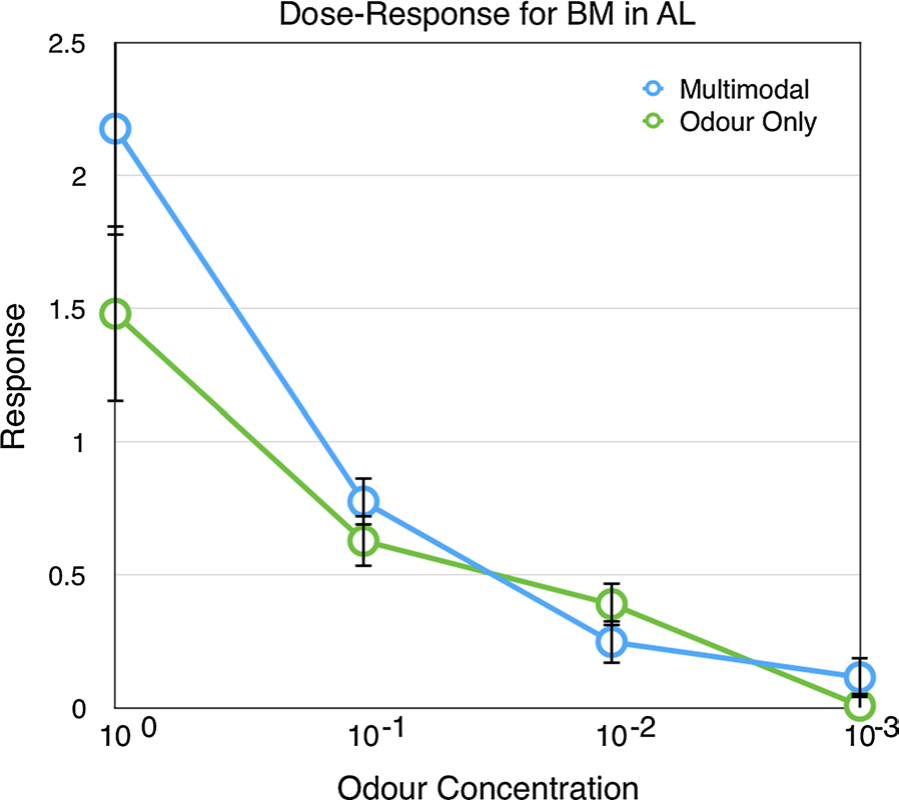
Responses of the antennal lobe to different odour concentrations (BM) with and without the colour stimulus (n $=$ 18). *Error bars* show standard error of mean

In experiment 1, the response magnitude for BM decreased in AL when the odour concentration was lowered (Linear regression, $r^2=0.5016, \hbox {P}<0.001$, Additional file 1). There was no differences for the responses to the odours with or without the colour stimulus present. (Fig. 1, P $=$ 0.353, 1, 1, and 0.26 for the four concentrations respectively, Mann–Whitney U test, Bonferroni corrected).

**Fig. 2:**
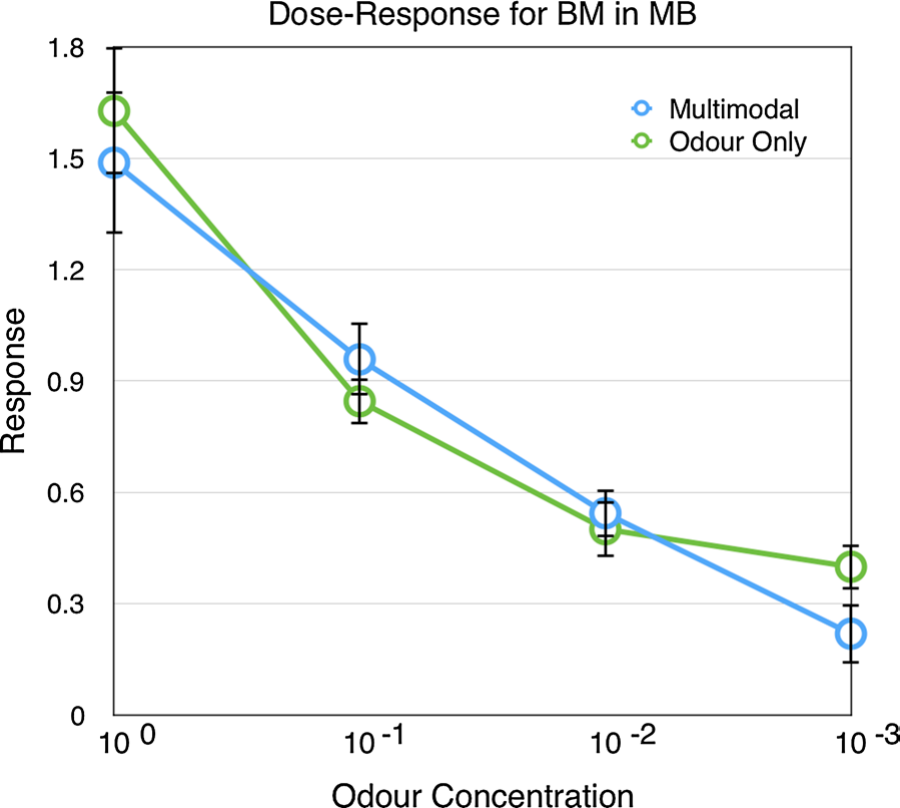
Responses of the mushroom body to different odour concentrations (BM) with and without the colour stimulus (n $=$ 21). *Error bars* show standard error of mean

Similarly, in experiment 2, the response of the MB also decreased with decreased odour concentration (Linear regression, $r^2=0.4498, \hbox {P}<0.001$, Additional file 2). There were no significant differences between the responses with or without colour for the different odour concentrations. (Fig. 2, P $=$ 1, 0.477, 1, and 1 for the four concentrations respectively, Mann–Whitney U test, Bonferroni corrected).

**Fig. 3:**
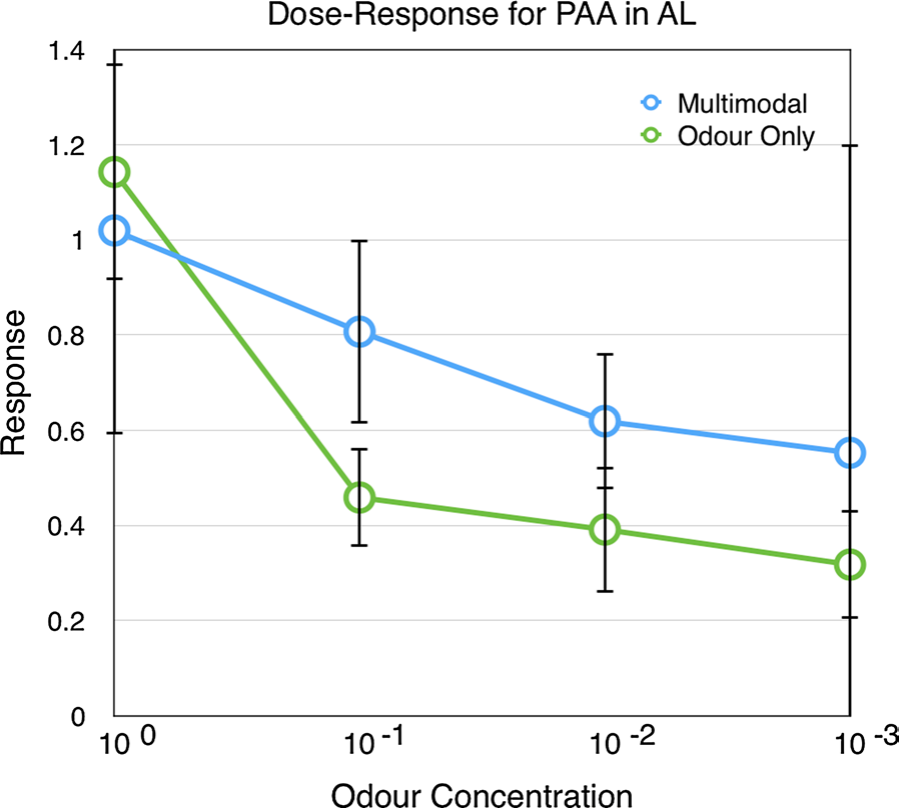
Responses of the antennal lobe to different odour concentrations (PAA) with and without the colour stimulus (n $=$ 9). *Error bars* show standard error of mean

**Fig. 4:**
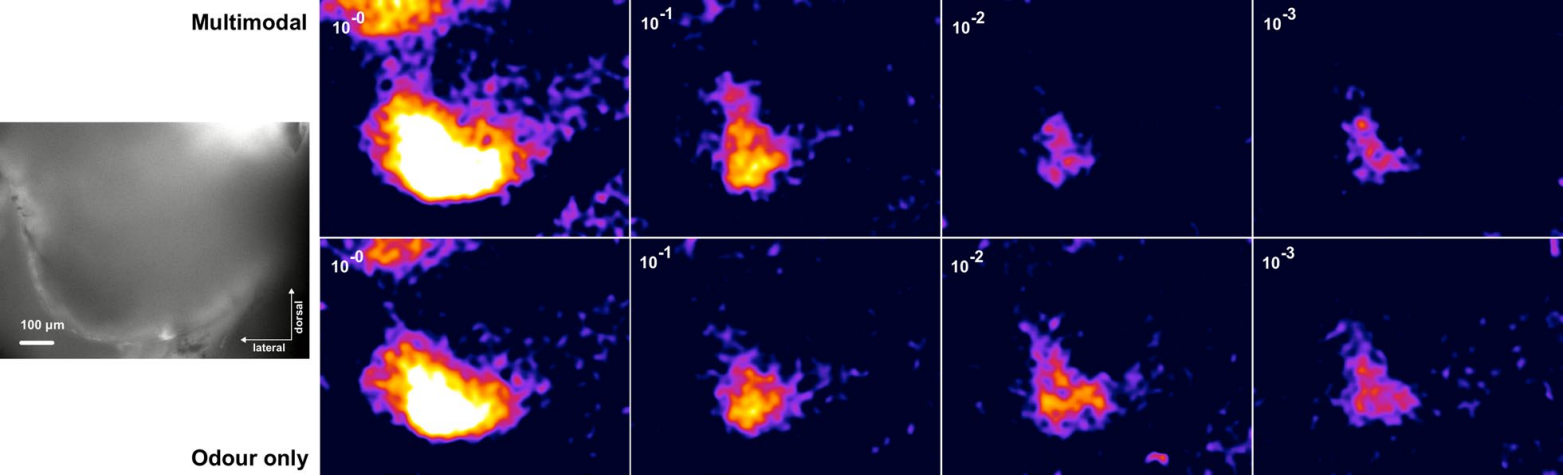
Activity patterns in AL example of the activity pattern in the antennal lobe with different odour concentration. *Top* Multimodal stimulus. *Bottom* Only odour

**Fig. 5:**
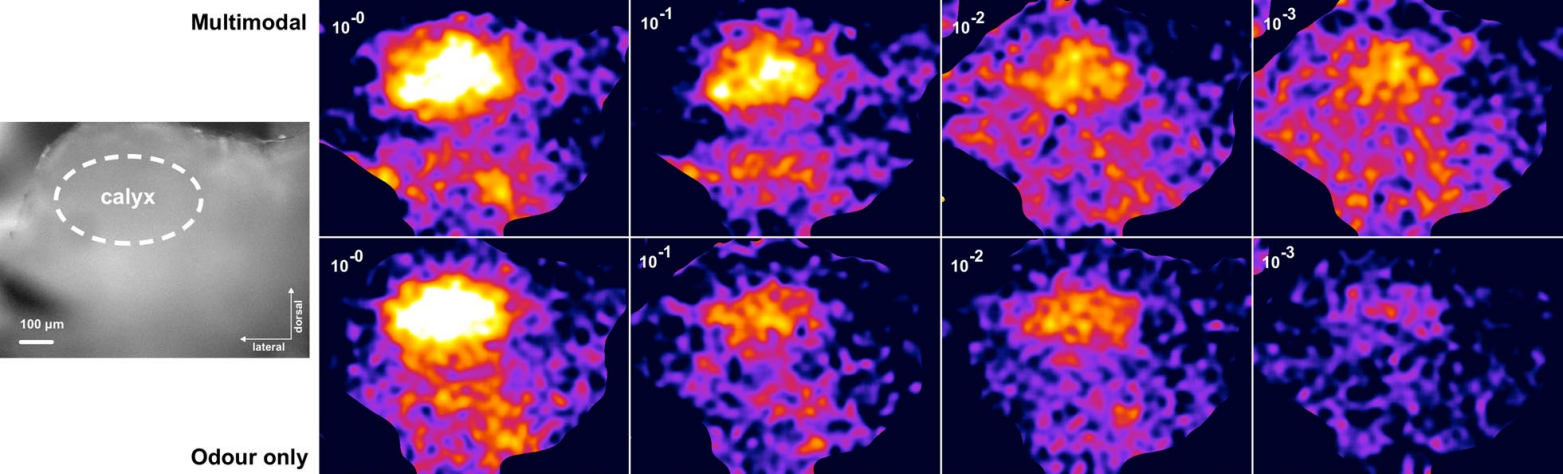
Activity patterns in MB Example of the activity pattern in the mushroom body with different odour concentrations. *Top* Multimodal stimulus. *Bottom* Only odour

### Visual cues enhances responses to a general flower odour in the mushroom body, but not int the antennal lobe

Experiment 3 and 4 used a general flower odour (Phenylacetaldehyde, PAA) in different concentrations with or without visual stimulation. The two modalities were presented together for 1 s while the responses of the AL or MB were recorded. Recordings in the Al were performed on 9 animals and for the MB recordings 22 animals were used.

In experiment 3, the response magnitude for PAA decreased in AL when the odour concentration was lowered (Linear regression, $r^2=0.12, \hbox {P}<0.01$, Additional file 3). Although the measured responses were higher for the multimodal stimuli, there were no significant differences between the responses to odour on its own and odour together with the visual stimulus (Figs. 3, 4, P $=$ 0.566, 1, 1, and 1 for the four concentrations respectively, Mann–Whitney U test, Bonferroni corrected).

**Fig. 6:**
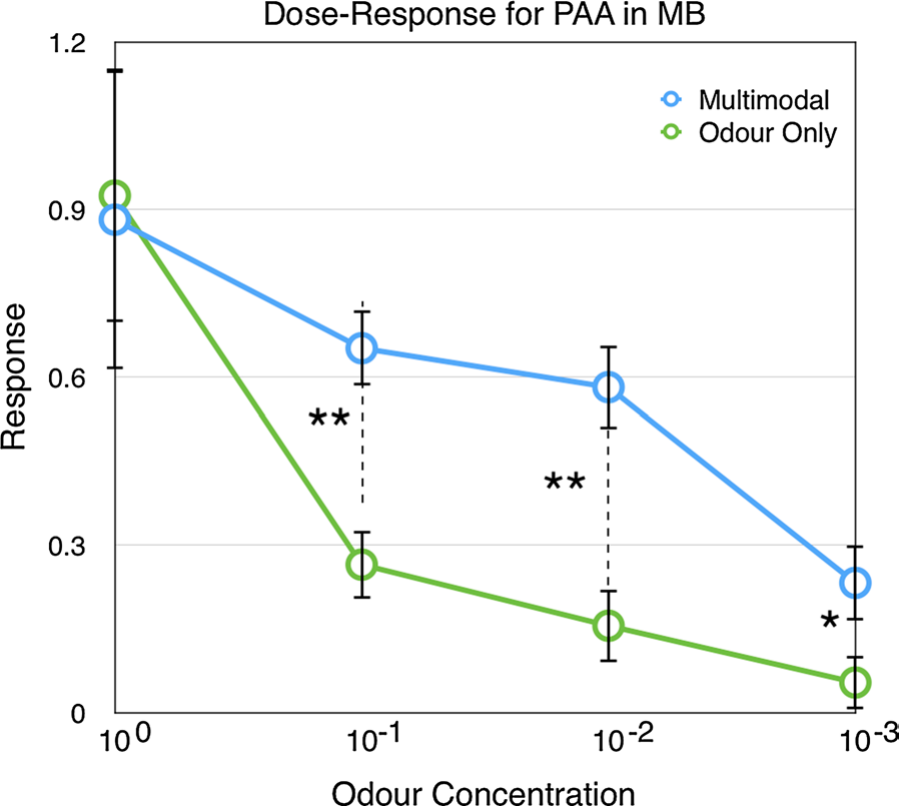
Responses of the mushroom body to different odour concentrations (PAA) with and without the colour stimulus (n $=$ 22). *Error bars* show standard error of mean

In experiment 4, the responses of the MB also decreased with decreased odour concentration (Linear regression, $r^2= 0.2709, \hbox {P}<0.001$, Additional file 4). There are clear responses to the multimodal stimulus in the MB (Fig. 5). In contrast to the recordings from the AL, the intensity of the responses in MB depended on whether the visual stimulus was present (Fig. 6). There is a significant increase in the response for all but the highest concentration ($\hbox {P}=0.295, {<}0.01, {<}0.01, {<}0.05$ respectively, Mann–Whitney U test, Bonferroni corrected)

### Multimodal interaction in the mushroom body shows *inverse effectiveness* for weaker stimuli

**Fig. 7:**
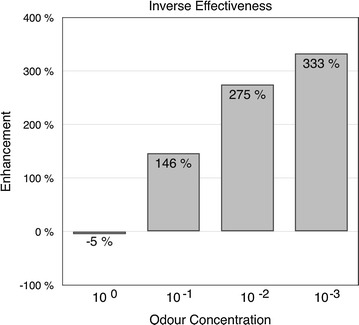
Inverse effectiveness of multimodal interaction in the *mushroom body* The multimodal enhancement is larger for the lower odour concentrations (n $=$ 22)

We calculated the degree of enhancement by the visual stimulus for the different odour concentrations for the data shown in Fig 6. The enhancement is defined as $E =(R_1-B)/(R_2-B)-1$, where $R_1$ and $R_2$ are the two responses to be compared and B is the background level when no stimulus is present. The enhancement clearly shows an inverse effectiveness for lower concentrations (Fig. 7). While there was very little change for the responses to the highest odour concentration, there were large enhancements for the lower concentrations ranging from a 146 % enhancement to 333 % for successively lower concentrations.

**Fig. 8:**
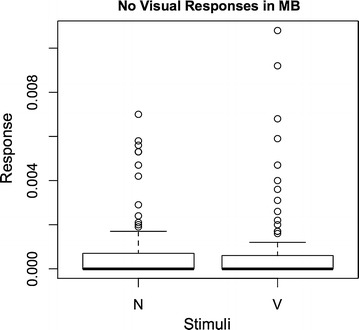
No responses to visual stimulus. The presentation of a visual stimulus (V) does not elicit a measurable response in the MB when compared to not stimulus at all (N) (n $=$ 94)

To exclude that the enhancement was due to a visual response that was added to odour response, we compared the responses of the MB to visual stimulus alone to MB activity with no stimulus present. There were no significant differences in the measurements when the visual stimulus was presented alone and when no stimulus was presented at all (P $=$ 0.96, Mann–Whitney U test, Fig. 8, Additional file 5).

### Visual cues do not influence the response latency in the mushroom body

**Fig. 9:**
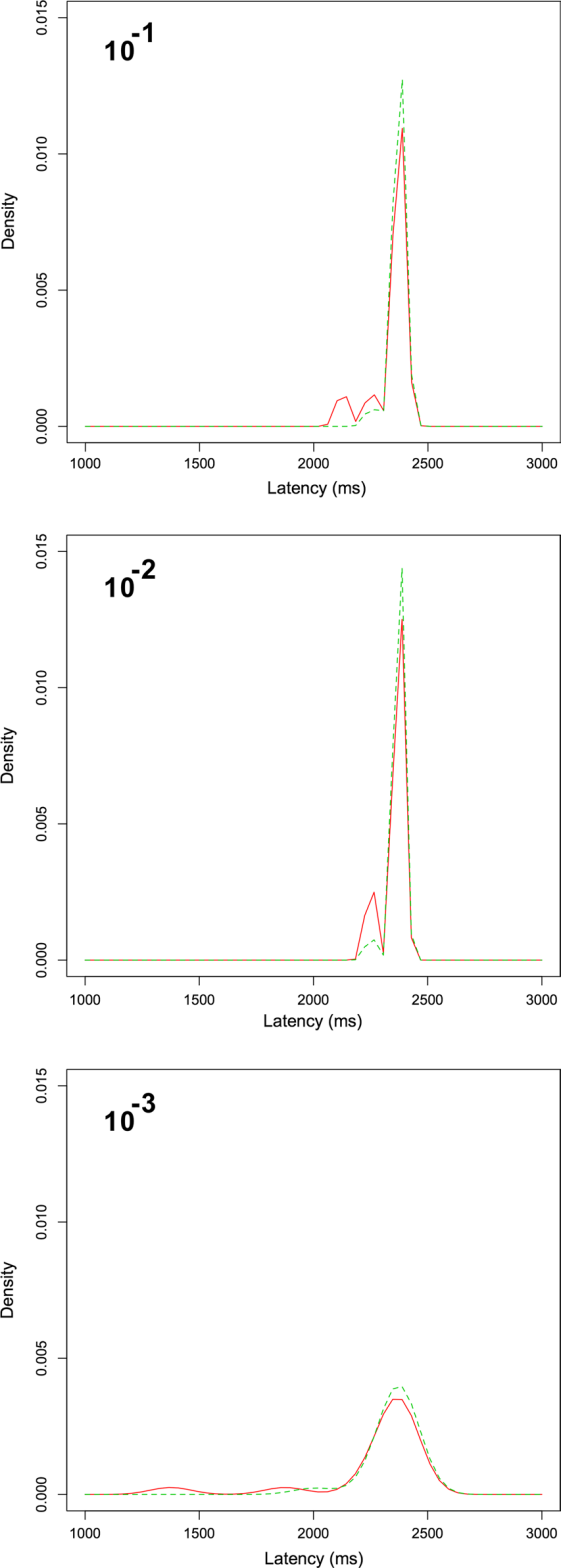
Response peak latency density plot of the latency of the response peak for three odour concentrations relative to the start of each trial for the data set in Fig. 6 (*red* multmodal, *green* odour only). There are no differences in the respons timing for any of the odour concentrations

We tested the latency until the start of the response for the odour stimulus compared to the multimodal stimulus. There were no significant differences (P $=$ 0.7244, Mann–Whitney U-test, Additional file 6). We also tested the latency of the peak of the response for the multimodal and unimodal odour stimuli (Fig. 9). There were no significant differences between the time of the peak for any of the odour concentrations (P $=$ 0.3505, 0.169, 0.2565, 0.2937, Mann–Whitney U-test, Additional file 7). This indicates that the multimodal processing occurs in real-time as the signals are received by the MB.

### Timing influences multimodal integration

**Fig. 10:**
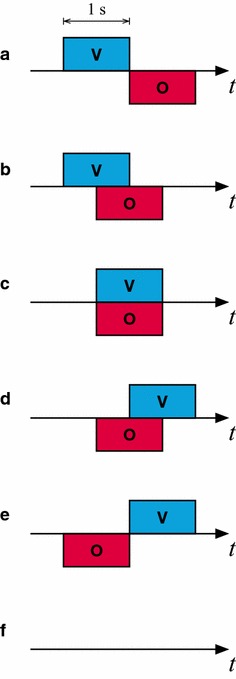
Stimulus timing five different temporal relations between the visual and the odour components of the multimodal stimulus stimulus used in the final experiment (**a**–**e**) and the blank stimulus (**f**)

The final experiment investigated the role of timing on the multimodal interaction. The visual stimulus was presented either before, together with, or after the odour stimulus (Fig. 10a, c, e). There were also two stimulus presentations where the two stimuli partially overlapped (Fig. 10b, d). In some trials (Fig. 10f), no stimulus was shown at all. The tests were made for the highest as well as the lowest odour concentration. The order of the different types of trials was balanced to avoid bleaching effects on successive trials. In total, recordings from 122 animals were used in the analysis.

**Fig. 11:**
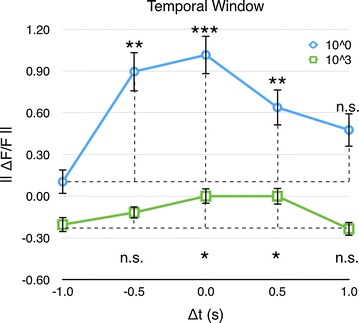
Temporal window the temporal window for multimodal interaction in the *mushroom body* for the highest (*blue*) and lowest (*green*) odour concentration (n $=$ 122). For both concentrations, the response was significantly enhanced, compared to the timing A, when the visual stimulus component coincided ($\Delta t = 0$ s) or came slightly after ($\Delta t = 0.5$ s) the odour. For the higher concentration, there was also an significant increase in the response when the visual stimulus preceded the odour with half a second ($\Delta t = -0.5$ s). The different temporal relations are shown in Fig. 10. *Error bars* show standard error of mean

For the highest odour concentration, the timing of the two components of the multimodal stimuli had an influence on the responses in MB (Kruskal–Wallis test, $\hbox {p}<0.001$, Fig. 11, Additional file 8). Looking at the difference between the individual temporal relations for the stronger odour concentration, there were a significant difference between the first timing (Fig. 10a) and timing b, c, and d but not for the last timing (a–b: $\hbox {p}<0.01$, a–c: $\hbox {p}<0.01$, a–d: $\hbox {p}<0.01$, a–e: P $=$ 0.156, Mann–Whitney U-test with Holm-correction).

For the lowest odour concentration, there was also a significant effect of the timing (Kruskal–Wallis test, P > 0.01, Fig. 11, Additional file 9). There was a significant increase in the response for timing c and d, but unlike for the highest concentration, there were no significant increase for timing b (a–b: P $=$ 0.936, a–c: $\hbox {P}<0.05$, a–d: $\hbox {P}<0.05$, a–e: P $=$ 0.974, Mann–Whitney U-test with Holm-correction, Fig. 11).

## Discussion

We investigated the multimodal responses of the brain of the moth *M. sexta* to stimuli consisting of odours and colours of different intensities in different temporal relationships to test *the principle of inverse effectiveness* and *the temporal window of multimodal integration*.

As expected, the responses of the antennal lobes decreases with lower odour concentration and these responses are not influenced by the visual stimulus (Figs. 1, 3). In contrast, the odour responses of the mushroom body were modulated by the presence and timing of the visual stimuli when the odour PAA was used.

The responses increased when visual stimulation occurred together with the odour, except for the strongest odour concentration (Fig. 6). This enhancement was stronger for the more diluted odourants (Fig. 7) showing an increased effectiveness of the multimodal interaction for the weaker stimuli. Non-linear interaction of this type is sometimes referred to as *synergistic interaction* [22] or *superadditivity* [9, 23, 24] of the stimuli.

At least two different ways to measure the enhancement has been suggested for spike count data [23]. One possibility is to compare the response to the multimodal stimulus to the maximum of the responses to the two unimodal stimuli [9]. The other alternative is to compare the multimodal response to the sum of the responses to the unimodal stimuli [24]. Since there is no measurable response in the MB to a unimodal visual stimulus (Figs. 8, [5]), the two methods to calculate the multisensory enhancement will give the same results for our data. However, these measures assume that there is no signal without a stimulus. Since this is typically not the case for imaging data, we modified the calculation to first subtract the background level.

Multimodal enhancement is often seen as a fundamental property of multimodal interaction, it is of importance to consider that a brain area can potentially perform multimodal processing without any signs of multimodal enhancement [8]. While multimodal enhancement is a sufficient condition for multimodal processing, it is not necessary [25]. Multimodal neurons may also perform additive or subadditive operations [25]. Such an effect has also been seen in the mushroom body of *M. sexta*, where some odours were suppressed by a visual cue rather than enhanced. The results are specific for the particular odours, their concentration and the colour stimulus used. For example, in two previous studies using a higher concentration of PAA, the same light stimulus used here instead produced suppression rather than enhancement [5, 18]. In the current study, there were a large difference between the responses for PAA and BM. While PAA was influenced by the visual stimulus, no such effect was seen for BM. Furthermore, the higher odour concentration in earlier studies also appeared to make the visual stimulus influence the on-set latency of the response [5]. No such effect was seen in this study.

Rowland and Stein proposed two possible models of multimodal interaction [26]. One possibility is that cross-modal signals are integrated directly when they arrive at the multimodal integration site. This is called *real-time integration*. The other possibility is that the integration takes place only after the individual signals have been received. This is called *delayed integration*. In the first case, we would expect the latency of the response peak to be the same for the unimodal stimuli and the multimodal response. In the second case, the latency of the multimodal response would be longer than that for the unimodal stimuli. When this was tested for the stimuli that produced differences between the unimodal and multimodal stimulation, there were no differences in the latency of the response peak to unimodal and multimodal stimuli (Fig. 9). This suggests that the interaction in the mushroom body constitutes a real-time interaction rather than delayed interaction. This is consistent with the results from multimodal interaction in the cat superior colliculus [26].

The timing of the visual and odour component influenced the interaction. The enhancement was optimal when the two stimulus modalities were presented together and decreased when the visual stimulus either preceded or succeed the odour with half a second (Figs. 10, 11). This indicates that there exists a temporal window within which the multimodal interaction is optimal. It also shows that sensory information can be integrated over time and does not need to reach the sensory organ of the animal at exactly the same time.

This result can be compared to behavioural experiments with *M. sexta*, where olfactory stimulation either before or after visually guided approach enhanced responsiveness to an odourless visual target [27]. Additionally, searching times were increased by either a transient olfactory stimulation before take-off or by having the flower model spatially separated from the odour source tracked by the moths [27]. The manipulation of floral cues showed that the feeding behaviour of *M. sexta* is based not only on the sensory stimulation per se but also on the temporal decoupling [27]. Olfactory stimulation before, during or after visual stimulation is sufficient to elicit probing. Thus, an odour plume can guide a moth to its source (the flower) when sustained, but it also can increase a moth?s responsiveness to a visual target when transient. Moths that approached the visual target in the absence of odour showed very low probabilities of proboscis extension, but this behaviour could be reversed by a transient odour puff administered as moths hovered in front of the flower model [27]. These studies are in line with our results that the responses are enhanced to multimodal stimuli.

Although we did not find any significant effect of the visual stimulus in the responses in the AL, there are recent results that suggest that the MB projects back to the AL in *Drosophila* [28] and honeybees [29], which could in principle support a changed odour sensitivity as a result of visual stimulation.

There are several extensions that could be made to the set-up. It would be useful to stimulate the two sides of the sensory systems of the animals separately, for example to stimulate only the left antenna or only the right eye. Another extension would be to use multi-fibre light guides to project visual patterns in addition to the different colours. This would make it possible to investigate the role of spatial location in multimodal integration.

## Conclusions

We have shown that odour and colour stimuli interact in the mushroom body of the insect over a wide range of odour concentrations. The interaction follows the same principles that can be found in other species: A stronger enhancement is found for weaker stimuli supporting that the insect brain follows the principle of *inverse effectiveness* for these stimuli. The results also show that the interaction follows a temporal rule, where the interaction is significantly stronger when the multimodal stimulus components at least partially overlap in time.

## Methods

### Animal preparation

The animals used were both males and females of the hawkmoth *M. sexta* (Lepidoptera: Sphingidae). Larvae were reared on an artificial diet modified from Bell and Joachim [30] with 200 mg beta-carotene/l added [31]. The animals were kept under a 16 h:8 h light/dark cycle at 23–25 $^\circ \hbox {C}$, and 40–50 % relative humidity. Experiments were performed on 2–4 days post-emergent naive moths.

Individual moths were secured in a plastic pipette, with the head protruding from the narrow end, and fixed by dental wax (Surgident, Heraeus Kulzer Inc). The head capsule was opened between the antenna and the eyes; muscle, glands, trachea, neural sheath and the oesophagus were removed to expose the AL and MB. A calcium-sensitive dye (calcium green-2-AM dye) was dissolved in 20 % Pluronic F-127 in dimethyl sulfoxide (Molecular Probes, Eugene, OR, USA), and diluted in moth Ringer solution to 30 mM, and then applied to the brain, leaving the preparation in a dark and cold (5–8 $^\circ \hbox {C}$) environment for 2 h.

### Multimodal stimulus generation

Stimulus generation and data collection was fully automatic and controlled by the TILL-vision 4.0 software (TILL Photonics) that could trigger colour and odour stimuli. The software allows the detailed control of the on-set and duration of each stimulus and makes it possible to produce different stimulus.

### Colour

The visual stimulus (V) was generated by a 3 mm LED. The intensity of the LED can be changed by the a custom made driver circuit and the produced spectrum can be modified by changing the LED. In the experiments described below, a LED with dominant wavelength at 430 nm was used and the intensity was set to approximately $0.01\hbox { cd/m}^2$. This blue colour is known to be attractive to moths during foraging [32, 33]. To not interfere with the optical recording, a fiber-optic light guide was used to transfer the visual stimulus to the eyes of the moth. The optically isolated light guides were docked to the eyes using small rubber tubes that were kept in place using dental wax. One LED and light guide was used for each eye.

### Odour

To produce the odour stimulus (O), the antennae were ventilated from a glass tube (7 mm internal diameter) with a continuous charcoal-filtered and moistened air stream (30 ml/s). The glass tube ended 10 mm from the antenna. The odourant was dissolved in paraffin oil and was applied on filter paper ($5\times 15$ mm) and inserted into a Pasteur pipette [34]. The pipette was in turn inserted trough a small hole in the continuous airflow glass tube with an air stream of 15 ml/s. Another air stream (5 m/s) was blown through the pipette by an automatically triggered puffer device (Syntech, Hilversum, The Netherlands) for 1 s into the continuous air stream. During odour stimulation, the air stream was switched from an empty pipette to an odour-laden one to minimise the influence of added air volume.

Odourants used were the plant-derived odour Bergamot (Aroma, essintial oil Citrus aurantium bergamia, Italy, BM) and phenylacetaldehyde (PAA) - odours that are known to elicit responses in the antennal lobes of *M. sexta* [34]. The dose was 50 $\upmu$g that was successively diluted by a factor of ten into four odour concentrations (indicated by $10^0, 10^{-1}, 10^{-2}$ and $10^{-3}$ in the figures).

### Optical recordings

Recordings were made in vivo after incubation and washing, using an Olympus microscope (10$\times$ objective NA 0.50; filter settings: dichroic 500 nm, emission LP 515 nm). The preparation was illuminated at 475 nm. Stimulation started at frame 12 and lasted 1 s. Images were binned (320 $\times$ 240 pixel) to increase signal-to-noise ratio. TILL LA PHOTONICS imaging software (Gräfeling, Germany) was used to record sequences of 38 frames (Experiment 1–4, 8 Hz, 80 ms exposure time) or 40 frames (Experiment 5, 8 Hz, 80 ms exposure time). The recorded image sequences were stored as 16 bit multi image TIFF files before they were analysed by the image processing software.

### Signal processing

First, noise was removed by a spatial Gaussian filter. Second, the response magnitude was calculated as the average $\Delta F/F$ for each frame, where *F* was estimated by comparing the signal in a sampling region to parts of the calcium fluorescence decay curve outside the potential response. Signals for each individual were normalized by dividing each measurement with the upper quartile or the distribution of the recorded signals for each animal. To generate images of the activity patterns (Figs. 4, 5), we used the linear method described by Balkenius et al. [35].

## Additional files

10.1186/s12868-016-0258-7  Dose-response data for Experiment 1.
10.1186/s12868-016-0258-7  ​Dose-response data for Experiment 2.
10.1186/s12868-016-0258-7  ​Dose-response data for Experiment 3.
10.1186/s12868-016-0258-7  ​Dose-response data for Experiment 4.
10.1186/s12868-016-0258-7  Data for measurements with visual cues or no stimulus.
10.1186/s12868-016-0258-7 Response latency data for Experiment 4.
10.1186/s12868-016-0258-7 Peak latency data for Experiment 4.
10.1186/s12868-016-0258-7  Data for different stimulus timings with higher odour concentration for Experiment 5.
10.1186/s12868-016-0258-7 Data for different stimulus timings with lower odour concentration for Experiment 5.

## Abbreviations

MB: mushroom body
AL: antennal lobe
*M. sexta*: *Manduca sexta*
BM: bergamot
PAA: Phenylacetaldehyde
E: enhancement
R1: response one
R2: response two
B: background
V: the visual stimulus
LED: light emitting diode
O: the odour stimulus
F: frame
